# Supplementary material for: The interactome of intact mitochondria by cross-linking mass spectrometry provides evidence for coexisting respiratory supercomplexes
Source: Mol Cell Proteomics. 2017 Dec 8;17(2):216–32. doi: 10.1074/mcp.RA117.000470 (PMC5795388; doi:10.1074/mcp.RA117.000470)
Supplement: Supplemental Data [file supp_RA117.000470_133922_0_supp_23978_fzffwf.zip › spectra_annotation/mito_DR_spectra_annotation/150-1-3-1-5-1.pdf]

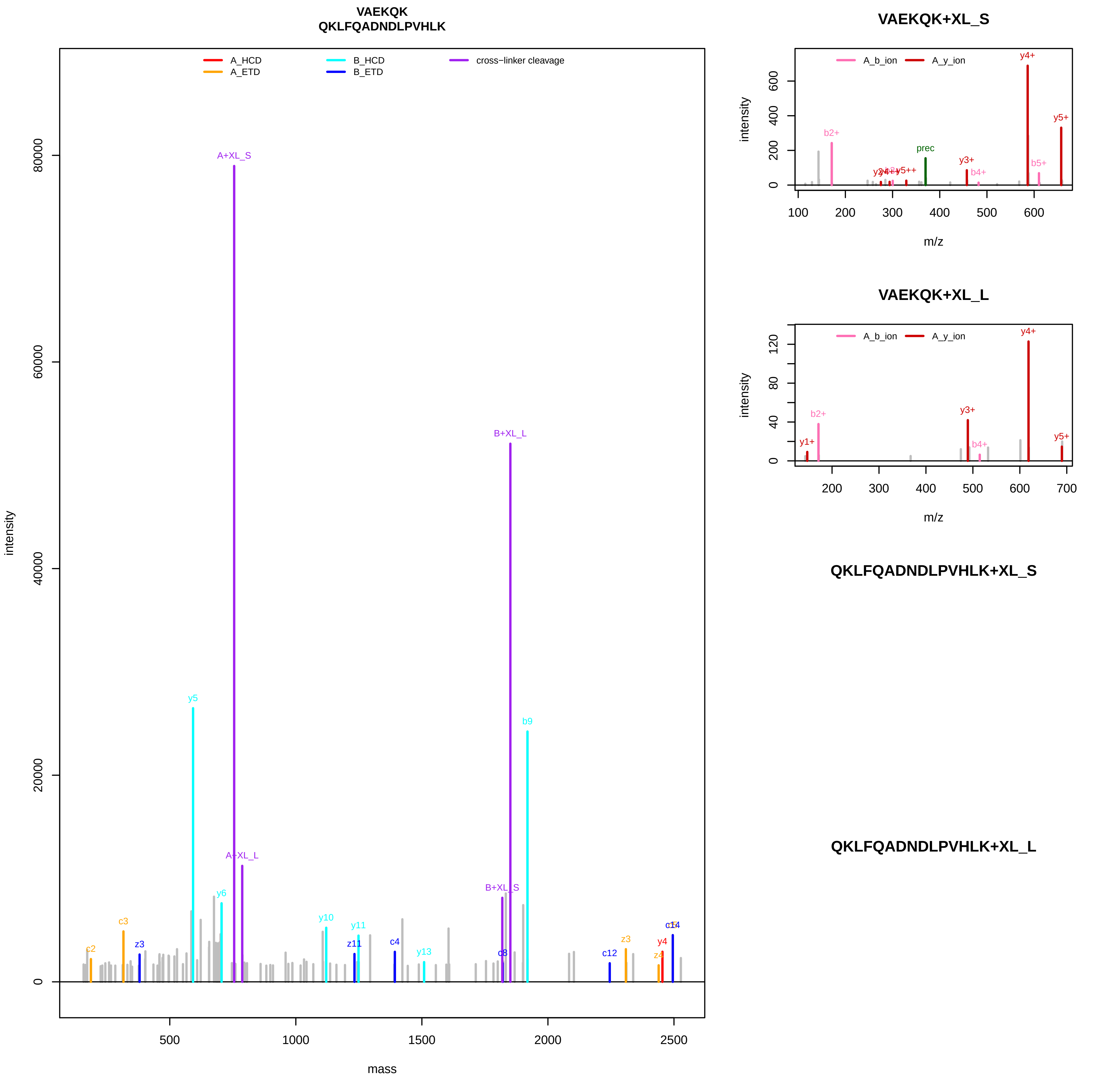
VAEKQK
QKLFQADNDLPVHLK
A_HCD
A_ETD
B_HCD
B_ETD
cross−linker cleavage
A+XL_S
B+XL_L
A+XL_L
B+XL_S
y5
b9
y6
y10
y11
y13
c2
c3
z3
z11
c4
c8
c12
z3
z4
y4
c14
intensity
mass
0
20000
40000
60000
80000
500
1000
1500
2000
2500
VAEKQK+XL_S
A_b_ion
A_y_ion
b2+
prec
y3+
b4+
y4+
b5+
y5+
y5++
intensity
m/z
VAEKQK+XL_L
A_b_ion
A_y_ion
y1+
b2+
y3+
b4+
y4+
y5+
intensity
m/z
QKLFQADNDLPVHLK+XL_S
QKLFQADNDLPVHLK+XL_L
